# An Unusual Occurrence of Multiple Metachronous and Synchronous Primary Cancers in a Female Patient

**DOI:** 10.1155/2020/5691732

**Published:** 2020-02-18

**Authors:** Tyler B. Torina, Emily L. Hudspeth, Joon Min Chun, William Zaloga, Carlisle Alderink, Yazan Abdeen

**Affiliations:** Mercy Hospital Fort Smith, Arkansas College of Osteopathic Medicine, USA

## Abstract

Multiple primary cancers, although uncommon, have been increasing in incidence in recent years. This trend is likely due to advances in factors such as diagnostic imaging, life expectancy, and interventional modalities with associated adverse effects. The patient presented in this case report is a 59-year-old Caucasian female with an extensive medical history who developed multiple primary cancers of the breast, mouth, gastrointestinal system, and lung in the span of four years. We attempted to elucidate the possible etiologies and risk factors that may have contributed. Given the patient's complex medical and social history, interventions, environmental, and genetic predispositions, it is impossible to pinpoint a single etiology. Rather, it is more likely that the interplay of multiple factors contributed to the findings of this case.

## 1. Introduction

Multiple primary cancers are described as the occurrence of multiple malignancies that develop from different tissues with distinct morphologies. Although each primary cancer allows for metastasis, multiple primary cancers are all independent in origin. These cancers are classified as metachronous or synchronous. Malignancies that occur within 6 months of the first primary tumor are defined as synchronous while those that develop after the first 6-month interval are defined as metachronous [1].

Incidence of multiple primary cancers was first cited in 1869 by Billroth (see [2]). Despite the advancements in medical knowledge since then, the etiology behind multiple primary cancers remains unclear. There is, however, research that suggests potential contributing factors such as exposure to radiotherapy, hormonal therapy, medical imaging, comorbidities, environmental factors, and genetic predispositions. This case report details the development of a patient's primary breast, oral, gastrointestinal, and lung cancers and the interplay of interventional, diagnostic, and psychosocial modalities.

## 2. Case History

The patient is a 59-year-old Caucasian female with an extensive medical history including obesity, depression, hypertension, hyperlipidemia, coronary artery disease, osteoporosis, chronic kidney disease, hyperplastic colon polyps, a back injury that required surgery, chronic obstructive pulmonary disease, and a smoking history of 50 pack years. This patient's family history is significant for lung cancer in both parents, although both were lifelong smokers.

**Figure 1:**
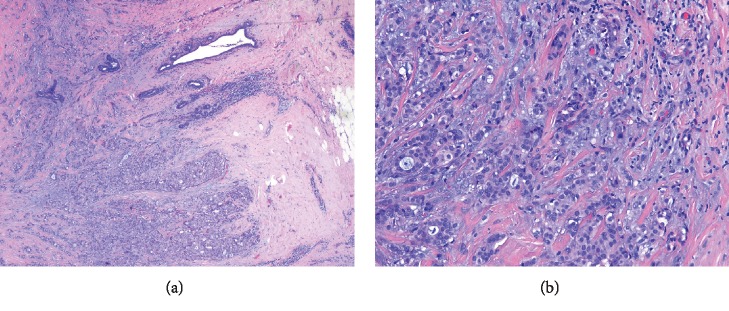
(a) T1cN0M0, Stage 1A, G1 left breast invasive ductal cell carcinoma under light microscopy (4x). (b) T1cN0M0, Stage 1A, G1 left breast invasive ductal cell carcinoma under light microscopy (20x).

The patient initially presented to her primary care physician with concern of a painful lump under her left nipple that was noticed 3-4 weeks prior upon self-examination. She rated the pain at 3 out of 10 (0-10 scale). Physical exam showed no skin changes, dimpling, or nipple discharge. Mammogram and ultrasound showed a 1.2 cm solid, irregular nodule that was compatible with breast carcinoma. With this finding, surgery consultation for biopsy was recommended. Lumpectomy with biopsy revealed sections of breast tissue containing tumor were composed of small, duct-like structures that infiltrated the stroma in a single-file pattern (Figure 1). The E-cadherin immunostain revealed staining in virtually all tumor cells which is consistent with a diagnosis of invasive ductal cell carcinoma of the breast. The patient elected for breast conservation treatment in which the surgeon subsequently excised larger margins of breast tissue surrounding the previously removed cancerous mass in addition to sentinel node excision and biopsy. All sentinel nodes were benign, and therefore, no staging scan was performed at this time. Genetic testing showed estrogen (ER+) and progesterone (PR+) receptors but negative for the human epidermal growth factor receptor 2 (HER2-) by fluorescence in situ hybridization (FISH), and a diagnosis of T1cN0M0, Stage 1A, G1 ER+/PR+/HER2- invasive ductal cell carcinoma was made. The patient received adjuvant radiotherapy, and a hormone-based drug, anastrozole, with regard to her genetic markers. Throughout treatment, the patient continued smoking and her preexisting depression worsened. Follow-up imaging after six months of treatment did not show recurrence.

**Figure 2:**
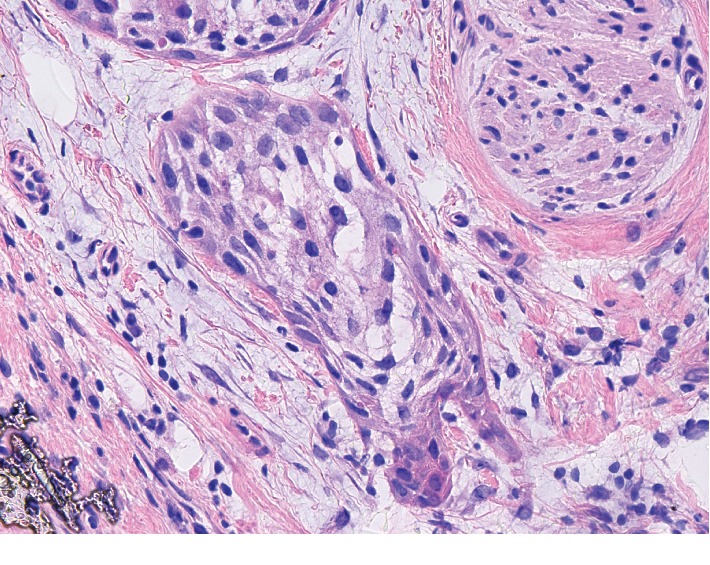
Photomicrograph (40x) of moderately differentiated squamous cell carcinoma of the mouth.

Just over a year after her breast cancer diagnosis, the patient presented with what she believed to be a tender ulcer on the right inferomedial margin of the tongue that had been present for 2-3 weeks and had been growing in size. She also complained of a tender knot on the right lateral side of her neck. The patient was referred to ENT for biopsy. A biopsy of the oral lesion on the floor of her mouth showed nests and clusters of squamous epithelial cells which infiltrated the underlying tissue. The cells were composed of irregular, hyperchromatic nuclei with large amounts of eosinophilic cytoplasm with scattered mitotic figures present (Figure 2). A diagnosis of moderately differentiated squamous cell carcinoma was made with the largest margins of the tumor measuring 1.6 × .8 cm. Oral surgery was recommended but the patient was reluctant due to financial hardships and personal reasons and wanted a second opinion. PET-CT scan from the base of the skull to mid-thigh was performed to follow up the oral lesion showed only local disease. However, the nuclear scan also revealed increased uptake in the lower right pelvic region. The mass was concerning given the patient's past surgical history of a total abdominal hysterectomy with bilateral salpingo-oophorectomy (TAH-BSO) in 2000 due to a precancerous cervical lesion. The possibility of ectopic ovarian tissue was initially considered, but CA-125 levels were normal. The patient was referred to OB/GYN and an ultrasound revealed a solid 7 cm mass. At this time, the patient elected surgical intervention for her oral cancer and underwent a mass palate excision with a right modified radical neck dissection and excision of the right submandibular gland. Five regional lymph nodes and tissue from the salivary gland were examined and benign.

**Figure 3:**
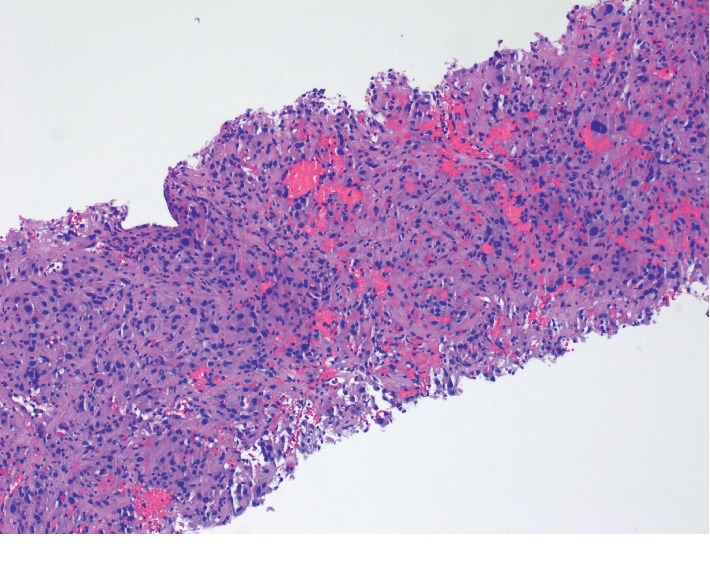
Photomicrograph (10x) of needle biopsy displaying pT3N0M0 gastrointestinal stromal tumor.

After her oral surgery, attention returned to her pelvic mass. An ultrasound-guided needle biopsy was performed. The needle biopsies contained sheets of cells which had oval hyperchromatic nuclei and large amounts of granular eosinophilic cytoplasm (Figure 3). Immunohistochemistry was positive for CD117 (c-KIT) and vimentin, focally positive for CD34, and negative for desmin and S-100 tumor cell markers. These pathological findings supported the diagnosis of a high risk (G2) gastrointestinal stromal tumor (GIST) of the jejunum and omentum. Soon after her GIST diagnosis, the patient underwent genetic testing for BRCA1, BRCA2, and other genetically relevant tumor markers, but all were negative. The patient was referred for colorectal surgery, and the pedunculated mass was removed by robotic resection. The tumor measured 7.4 × 5.3 × 4.2 cm with invasion into the muscularis propria, serosa, mesenteric fat, and omentum, but with no vascular or lymphatic invasion. She was then treated with Gleevec 400 mg daily with a goal of continued treatment for the next three years. At this juncture, the patient had metachronous (breast, oral, and GIST) and synchronous (oral and GIST) multiple primary cancers.

A year after her oral surgery, the patient returned for a restaging PET-CT scan for her oral, breast, and GIST tumors which showed increased uptake in the oral cavity only. Biopsy was performed and the results were benign. The patient continued her hormonal therapy and Gleevec with routine follow-ups.

**Figure 4:**
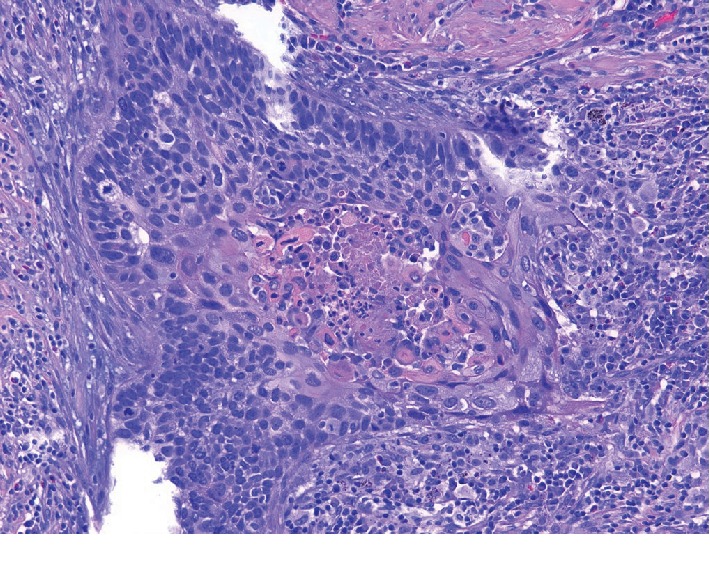
Photomicrograph (20x) Stage 1 poorly differentiated squamous cell carcinoma of the lung.

One year later, and two years since her oral cancer diagnosis, the patient presented with worsening fatigue, weakness, and diarrhea. She also complained of new onset nausea, abdominal pain, decreased appetite, and weight loss. In light of these symptoms, a restaging CT scan was ordered which incidentally showed a pulmonary nodule in the right inferior lobe. Needle biopsy of the nodule showed a tumor composed of nests and clusters of cells which had hyperchromatic nuclei and scanty cytoplasm with numerous mitotic figures (Figure 4). Immunoperoxidase stains were positive for cytokeratin and P40 and negative for TTF1 and synaptophysin. These findings led to a diagnosis of Stage 1 poorly differentiated squamous cell carcinoma of the lung. The patient underwent a brain MRI and follow-up PET-CT scan to rule out metastasis and both scans were negative. Sublobar resection and mediastinal lymphadenectomy were recommended given the patient's COPD and location and isolation of the 2.8 cm lesion. Surgical margins and regional lymph nodes were benign.

**Figure 5:**
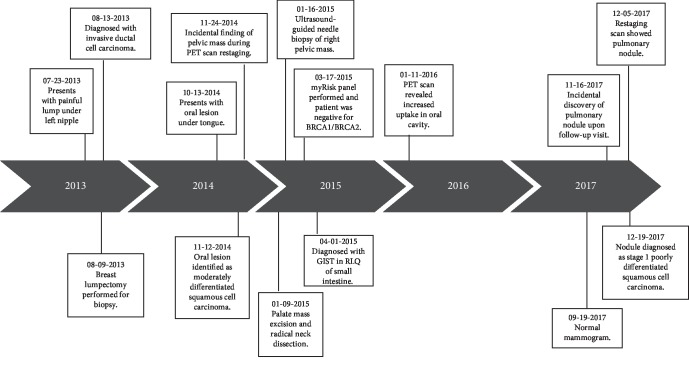
Timeline showing patient's progression.

The patient is still currently receiving adjuvant hormone therapy for her breast cancer with the goal of continued treatment for five years. She is tolerating this treatment well. She is also being followed up with ENT for her oral cancer and has had no recurrence. She is still being treated with Gleevec daily and continues to receive follow-up scans for her GIST. Her lung cancer status is being followed with CT scans every three months. A detailed timeline is given which illustrates the rapid development of each cancer in a short period of time (Figure 5).

## 3. Discussion

Research has indicated that individuals with one primary cancer are more likely to develop a second primary cancer compared to the general population. While the reason for this is still unknown, incidences of multiple primary cancers in general have been increasing in recent years [3]. This trend might be attributed to earlier detection due to improved imaging capabilities and increased survival rates due to improved therapeutic measures [4, 5]. Additionally, intrinsic risk factors for cancer associated with radiotherapy, hormonal therapy, medical imaging, environmental factors, and genetic disposition should be considered.

Extended radiotherapy has been shown to be associated with the development of second primary cancers. Regions that are anatomically near the targeted treatment area are more at risk for the development of malignancy. Specifically, an increased incidence of lung cancers has been noted following radiotherapy of the breast region [3]. Additionally, patients exposed to radiation from CT scans and mammograms have an increased incidence of developing cancer [6]. It is estimated that CT use is attributable to as much as 2% of all cancers in the United States [7]. While this statistic is not alarmingly high, it is possible that extensive use of medical imaging could potentiate multiple primary cancers in individuals with numerous cancer risk factors. This patient had a multitude of imaging performed throughout her course of treatment in addition to a dense medical history with imaging performed not noted in this case report. More research is obviously needed on these fronts in order to include these suggestions as definite causes for this patient's presentation.

The effects of smoking tobacco on cancer development is well documented and is one of the most avoidable risk factors as it leads to the initial mutation of and further proliferation of normal cells [8]. Some notable mutations include K-RAS and p53 genes and result from DNA damage due to benzopyrenes, polycyclic aromatic hydrocarbons, and other harmful substances found within tobacco smoke [9]. Other studies have even hypothesized that nicotine is responsible for promoting angiogenesis and tumor growth [10]. Although the complete effect and understanding of tobacco smoke on cancer has yet to be elucidated, it has been associated with at least 17 types of human cancer including lung squamous cell, oral cavity, esophagus, colorectal, and stomach cancers [8]. Thus, it is reasonable to conclude that smoking may have contributed to some of the cancers seen in this patient. Particularly, the primary cancers that developed after the initial breast cancer may have been more so affected by cigarette smoke given the time of onset. One study demonstrated that amongst smokers that developed second primary tumors, those who quit had an 11.55-year gap before the second tumor manifested. In smokers who did not quit, the gap before a new tumor was significantly less at 6.10 years [11]. This patient developed multiple new primary tumors within 4 years while continuing to smoke tobacco after the discovery of the initial breast cancer.

The depression which was diagnosed after the onset of breast cancer is another consideration for the development of subsequent primary tumors. Depression can lead to neuroendocrine, metabolic, and behavioral changes such as increased smoking, alcohol, and excessive calorie intake that increase the risk of cancer [12]. Studies have also linked depression with chronic inflammation with increases in IL-1, IL-6, and C-reactive protein which also increases the risk of cancer [11]. However, it should be noted that the relationship between depression and cancer risk is controversial. While some studies suggest that depression is a risk factor [13], others have found no association [14]. Meta-analysis on currently available epidemiologic evidence of depression as a risk factor for cancer shows a small positive association between depression and occurrence risk of overall cancer with moderate heterogeneity between studies analyzed [15]. However small, given the unusual number of primary tumors present, depression as a risk factor is worth considering.

Lastly, genetic disposition is important to consider when looking at the etiology of multiple primary cancers. For example, there is evidence that suggests genetic disposition as a contributing factor to lung cancer [16]. One study found that 13.7% of more than 26,000 patients diagnosed with lung cancer had one or more first-degree relatives who had also been diagnosed [16]. Since the patient described here had a familial history of lung cancer, it is reasonable to conclude she was at a high risk for its development. Germline mutations can also play a role in the development of multiple primary cancers. Hereditary cancer syndromes, such as those caused by germline mutations, account for approximately 5% of all cancers [17]. For example, the Li-Fraumeni syndrome is a mutation in the TP53 tumor suppressor gene, which causes a high likelihood that these individuals will develop histologically distinct tumors throughout their life. While this syndrome is most associated with breast cancer, sarcomas, leukemia, and brain tumors, it has an extremely broad effect and can cause a number of various malignancies [18]. Therefore, we must consider the possibility that a similar germline mutation could have contributed to the development of multiple primary cancers in this patient.

Treatment of multiple primary cancers is complicated and requires coordination between providers across multiple specialties. It also requires informed participation by the patient in order to adapt to unforeseen developments. Other documented cases of localized multiple primary tumors have been managed with surgery or radiation therapy covering each malignancy [19, 20]. As noted in this case report, this patient's multiple primary tumors have been managed similarly thus far. Going forward, she will continue to receive adjuvant hormone therapy for breast cancer. Her oral cancer will continue to be monitored by ENT. Her GIST will be managed with continuation of Gleevec daily and routine follow-up scans. Her lung cancer will also be followed with CT scans every three months. As treatment modalities improve, it is likely that the chance of a patient surviving long enough to develop additional primary tumors will increase.

Given the complex nature of presentation of multiple primary cancers in this patient, it is impossible to pinpoint a single risk factor that was responsible. Rather, it is likely that multiple aspects of the patient's social and medical histories contributed to these developments. While we attempted to discuss the major contributing factors according to the available research, there are still details that current scientific knowledge is unclear on that might help further elucidate the findings of this case. Although inquiry into the epidemiological and clinical perspectives of such cases is rising, definitive statements have remained elusive given the complex interplay of social and medical factors involved. This case study serves as another data point in leading to a more clear understanding of management and prognosis regarding multiple primary tumor occurrences in a single patient.

## References

[1] Moertel C. G., Dockerty M. B., Baggenstoss A. H. (1961). Multiple primary malignant neoplasms. I. Introduction and presentation of data. *Cancer*.

[2] Pennell V. (1958). Primary carcinoma multiplex. a series of 17 cases with review of the literature. *The British Journal of Surgery*.

[3] Owen L. J. (1921). Multiple malignant neoplasms. *Journal of the American Medical Association*.

[4] American Cancer Society *Cancer facts & figures 2016*.

[5] Supramaniam R. (2008). New malignancies among cancer survivors: SEER cancer registries, 1973–2000. *Journal of Epidemiology and Community Health*.

[6] Lin E. (2010). Radiation risk from medical imaging. *Mayo Clinic Proceedings*.

[7] Brenner D., Hall E. (2007). Computed tomography--an increasing source of radiation exposure. *The New England Journal of Medicine*.

[8] Alexandrov L. B., Ju Y. S., Haase K. (2016). Mutational signatures associated with tobacco smoking in human cancer. *Science*.

[9] Hecht S. S. (2012). Research opportunities related to establishing standards for tobacco products under the Family Smoking Prevention and Tobacco Control Act. *Nicotine & Tobacco Research*.

[10] Heeschen C., Jang J. J., Weis M. (2001). Nicotine stimulates angiogenesis and promotes tumor growth and atherosclerosis. *Nature Medicine*.

[11] Howren M. B., Lamkin D. M., Suls J. (2009). Associations of depression with C-reactive protein, IL-1, and IL-6: a meta-analysis. *Psychosomatic Medicine*.

[12] Luppino F. S., de Wit L. M., Bouvy P. F. (2010). Overweight, obesity, and depression: a systematic review and meta-analysis of longitudinal studies. *Archives of General Psychiatry*.

[13] Huang T., Poole E. M., Okereke O. I. (2015). Depression and risk of epithelial ovarian cancer: results from two large prospective cohort studies. *Gynecologic Oncology*.

[14] Archer G., Pikhart H., Head J. (2015). Do depressive symptoms predict cancer incidence?: 17-year follow-up of the Whitehall II study. *Journal of Psychosomatic Research*.

[15] Jia Y., Li F., Liu Y. F., Zhao J. P., Leng M. M., Chen L. (2017). Depression and cancer risk: a systematic review and meta-analysis. *Public Health*.

[16] Bailey-Wilson J. E., Amos C. I., Pinney S. M. (2004). A major lung cancer susceptibility locus maps to chromosome 6q23-25. *American Journal of Human Genetics*.

[17] Harper P. (2004). *Practical Genetic Counselling*.

[18] Rahner N., Steinke V. (2008). Hereditary cancer syndromes. *Deutsches Ärzteblatt International*.

[19] Heroiu Cataloiu A. D., Danciu C. E., Popescu C. R. (2013). Multiple cancers of the head and neck. *Maedica*.

[20] Zhang Z., Gao S., Mao Y. (2016). Surgical outcomes of synchronous multiple primary non-small cell lung cancers. *Scientific Reports*.

